# H9c2 Cardiomyocytes under Hypoxic Stress: Biological Effects Mediated by Sentinel Downstream Targets

**DOI:** 10.1155/2021/6874146

**Published:** 2021-09-30

**Authors:** Mariarosaria Boccellino, Giovanni Galasso, Pasqualina Ambrosio, Paola Stiuso, Stefania Lama, Erika Di Zazzo, Sonia Schiavon, Daniele Vecchio, Luca D'ambrosio, Lucio Quagliuolo, Antonia Feola, Giacomo Frati, Marina Di Domenico

**Affiliations:** ^1^Department of Precision Medicine, University of Campania “Luigi Vanvitelli”, Naples, Italy; ^2^Department of Medico-Surgical Sciences and Biotechnologies, Sapienza University of Rome, Latina, Italy; ^3^Department of Biology, University of Naples “Federico II”, Naples, Italy; ^4^Department of Medical-Surgical Sciences and Biotechnologies, Sapienza University of Rome, Italy; ^5^IRCCS Neuromed, Pozzilli, Italy; ^6^Department of Biology, College of Science and Technology, Temple University, Philadelphia, PA, USA

## Abstract

The association between diabetes and cardiovascular diseases is well known. Related diabetes macro- and microangiopathies frequently induce hypoxia and consequently energy failure to satisfy the jeopardized myocardium basal needs. Additionally, it is widely accepted that diabetes impairs endothelial nitric oxide synthase (eNOS) activity, resulting in diminished nitric oxide (NO) bioavailability and consequent endothelial cell dysfunction. In this study, we analyzed the embryonic heart-derived H9c2 cell response to hypoxic stress after administration of a high glucose concentration to reproduce a condition often observed in diabetes. We observed that 24 h hypoxia exposure of H9c2 cells reduced cell viability compared to cells grown in normoxic conditions. Cytotoxicity and early apoptosis were increased after exposure to high glucose administration. In addition, hypoxia induced a RhoA upregulation and a Bcl-2 downregulation and lowered the ERK activation observed in normoxia at both glucose concentrations. Furthermore, a significant cell proliferation rate increases after the 1400 W iNOS inhibitor administration was observed. Again, hypoxia increased the expression level of myogenin, a marker of skeletal muscle cell differentiation. The cardiomyocyte gene expression profiles and morphology changes observed in response to pathological *stimuli*, as hypoxia, could lead to improper ventricular remodeling responsible for heart failure. Therefore, understanding cell signaling events that regulate cardiac response to hypoxia could be useful for the discovery of novel therapeutic approaches able to prevent heart diseases.

## 1. Introduction

The damage induced by ischemia is related to energy failure production to satisfy the basal needs of the jeopardized myocardium. In the myocardium, during ischemia, the carbohydrate aerobic oxidation is compromised, so the anaerobic metabolism of exogenous glucose becomes a relevant strategy to generate ATP [1, 2]. Diabetic patient's cardiomyocytes show an idiopathic primary defect in glycolysis induction, characterized by a glucose reduction intake and utilization [3].

The association between diabetes and cardiovascular and cerebrovascular diseases is well known [4, 5]. Diabetes is strongly associated with an increased incidence of heart failure, directly promoting cardiac hypertrophy, fibrosis, and apoptosis. These changes, in turn, contribute to the development of ventricular dysfunction [6]. Cardiomyocytes synthetize and secrete adiponectin, an adipocytokine that regulates glucose and fatty acid metabolism and exerts antioxidant and anti-inflammatory effects [7, 8], to protect against myocardial ischemic and reperfusion injuries [9]. In addition, it is widely accepted that in both type 1 and type 2 diabetes, there is an endothelial nitric oxide synthase (eNOS) activity impairment, resulting in diminished nitric oxide (NO) bioavailability and consequently in vascular alterations. Although eNOS responses are reduced in congestive heart failure, ischemic preconditioning induces iNOS expression, resulting in high and sustained levels of NO. The NO oxidizes myoglobin, thus decreasing mitochondrial oxygen supply, and inhibits mitochondrial respiration [10, 11].

In response to pathological *stimuli* such as biomechanical stress resulting from hypoxia, cardiomyocytes modify their morphology, increase protein synthesis, and reactivate cardiac fetal genes [12, 13]. These changes eventually become maladaptive, leading to unfavorable ventricular remodeling and heart failure through aberrant activation of signaling pathways. Therefore, the understanding of cell signaling events that regulate cardiac function can facilitate therapeutic approaches to prevent heart diseases [14].

G protein coupled receptor (GPCR) and tyrosine kinase receptor (RTK), by activating ERK1/2, could be involved in the mitigation of hypertrophic cardiomyopathy treatment [15–24]. In myocardial hypertrophy and heart failure, Rho and Rac are implicated in signalling pathways that regulate various cellular functions, such as actin stress fiber assembly and focal adhesions [25–28]. In particular, RhoA plays a pivotal regulatory role in motility, proliferation, and differentiation of cardiomyocytes in response to stress conditions [29–34]. Together with RhoA, Rac1 represents the most characterized member of small G proteins in myocardial signaling. Rac1 regulates hypertrophic remodeling of cardiomyocytes that is related to the MAPK cascade activation [35, 36]. The mechanical stretch, reoxygenation damage, and ischemia/reperfusion injury promote ROS formation that is linked to Rac1 activation [37]. Indeed, the Rho kinase pathway activation affects cardiomyocytes and leads to inflammatory and proliferative changes in blood vessels. In this study, we want to analyze the response of the embryonic heart-derived H9c2 cells to hypoxia after acute administration of high glucose concentration to elucidate the role of glucose in the cell response to hypoxia, investigating the cell proliferation, apoptosis, oxidative stress, and molecular pathways modulated.

## 2. Materials and Methods

### 2.1. *In Vitro* Cell Culture Studies

Rat cardiomyocyte (H9c2) (ATCC, Manassas, VA) cells were cultured in DMEM supplemented with 10% fetal bovine serum, 100 U/ml of penicillin, and 100 mg/ml of streptomycin at 37°C in a 5% CO_2_ humidified atmosphere. Cell culture media was changed every 2–3 days, and cells were subcultured once they reached 70–80% of confluence [38].

### 2.2. Cell Proliferation Assay

Cell viability was assessed by the MTT [3-(4,5-dimethylthiazolyl)-2,5-diphenyl tetrazolium bromide] assay as described [39, 40]. Briefly, 3 × 10^3^ cells were seeded in 96-well plates and pretreated with four different concentrations of glucose (8, 16, 32, and 64 mM) for 30 min or 15 min, and then placed in a hypoxia chamber for 24 h (data not shown). MTT solution at 10% was added to each well and incubated for 2 h. Then, the excess medium was removed, and 100 *μ*l of a solution of 1 N hydrochloric acid was added to dissolve the formazan crystals. The mixture was shaken for about 20 min, and the optical density in each well was measured using a microplate spectrophotometer (Microplate Reader Model 550, Bio-Rad, California, USA) at 570 nm. Triplicate experiments were performed for each condition. The cell viability percentage (%) was calculated by comparison with the corresponding control. On the basis of results obtained, 8 mM and 32 mM were chosen to reproduce normoglycemic and hyperglycemic conditions, respectively. For analysis of the effect of EHT 1864 [5-(5-(7-(trifluoromethyl)quinolin-4-ylthio)pentyloxy)-2-(morpholinomethyl)-4H-pyran-4-one dihydrochloride], which inhibits Rac1 function, we preincubated H9c2 cells with EHT 1864 (20 *μ*M) for 30 min. Subsequently, the cells were further incubated in the presence of glucose (8 and 32 mM).

### 2.3. Hypoxic Stress

To induce a hypoxic stress condition, 3 × 10^3^ H9c2 cells were plated in a sealed humidified chamber (Billups-Rothenburg, Del Mar, California) supplied with 5% carbon monoxide and 95% nitrogen for 24 h. Finally, the MTT assay was performed to test cell viability and the hypoxic threshold in cardiomyocytes.

### 2.4. Apoptosis Assay Using Annexin V-FITC/PI Flow Cytometric Method

Apoptotic cells were detected by annexin V-FITC/PI staining assay following the manufacturer procedure (V13242; Thermo Fisher) [41–43]. The cells were washed twice with annexin V-binding buffer (140 mM NaCl, 10 mM HEPES, and 2.5 mM CaCI2, pH 7.4), resuspended in 1 ml of the same buffer, and incubated in ice for 30 min with 2 l of 140 nM annexin V-FITC. Five minutes before flow cytometry analysis, 5 l of PI (50 g/ml H_2_O stock solution) was added to each sample and then analyzed by flow cytometry (Becton Dickinson, San Jose, CA).

### 2.5. Western Blot Analysis

H9c2 cells were lysed at 4°C for 1 h in a lysis buffer (50 mM Tris-HCl pH 7.5, 150 mM NaCl, 1% Triton X-100) supplemented with a cocktail of phosphatase and proteinase inhibitors (1 mM sodium orthovanadate, 1 mM phenylmethylsulfonyl fluoride (PMSF), 10 mg/ml leupeptin, 10 mg/ml pepstatin, and 10 mg/ml aprotinin) as described elsewhere [44]. After centrifugation of the lysates at 13,000 × *g* for 10 min, the supernatants were quantified for protein content by the Bradford method. Aliquots containing 30 *μ*g of protein per lane were subjected to SDS-10% PAGE under reducing (5% *β*-mercaptoethanol) conditions and electroblotted onto nitrocellulose membrane filters (Auricchio et al., 1995). The blots were blocked with 5% nonfat milk in 20 mM Tris-HCl, pH 7.5, 500 mM NaCl plus 0.1% Tween (TBS-T). The membranes were subsequently incubated overnight at 4°C in agitation with appropriate primary antibodies. Rabbit polyclonal anti-MyoD (sc-304 (C-20); Santa Cruz) and rabbit polyclonal anti-myogenin (sc-576; Santa Cruz) antibodies were used to detect MyoD and myogenin, respectively. Mouse monoclonal anti-RhoA (sc-418; Santa Cruz Biotechnology, Inc.) and anti-Rac1 (#17-283; Millipore) antibodies were used to detect RhoA and Rac1, respectively. p85*α*PI3K (SC-1637, Santa Cruz Biotechnology), ERK2 (C-14), (sc-154; Santa Cruz Biotechnology), and p-ERK (E-4) (sc-7383; Santa Cruz Biotechnology, Inc.) antibodies were used to detect p85*α*PI3K, ERK2, and p-ERK, respectively. Tubulin was detected using mouse monoclonal anti-tubulin antibody as elsewhere described (Sigma-Aldrich) [45, 46]. Immunoreactive proteins were revealed using the ECL system (GE Healthcare).

### 2.6. Morphological Evaluation of Cardiomyocytes by Confocal Microscopy

H9c2 cells were seeded on glass slides for 24 h as previously described [47]. After the different experimental treatments, cells were fixed for 20 min in 3% (*w*/*v*) paraformaldehyde (PFA) solution and permeabilized for 10 min with 0.1% (*w*/*v*) Triton X-100 in phosphate-buffered saline (PBS) at room temperature. To prevent nonspecific interactions of antibodies, cells were treated for 2 hr in 5% fetal bovine serum (FBS) in PBS, then cells were incubated with different specific mouse monoclonal antibodies (anti-vimentin, anti-RhoA) in blocking solution and 3% (*w*/*w*) BSA in TBS-Tween 0.1% for 2 hr at 37°C. After multiple washes, cells were incubated with a specific secondary antibody diluted 1 : 1000 in blocking solution for 1 hr at room temperature. The slides were mounted on microscope slides by Mowiol medium. The analyses were performed with a Zeiss LSM 510 microscope equipped with a plan-apochromat objective X63 (NA 1.4) in oil immersion. Vimentin fluorescence was collected in a multitrack mode.

## 3. Results and Discussion

### 3.1. Hypoxic Injury Effects on H9c2 Cell Viability after Acute Exposure to High Glucose Levels

**Figure 1:**
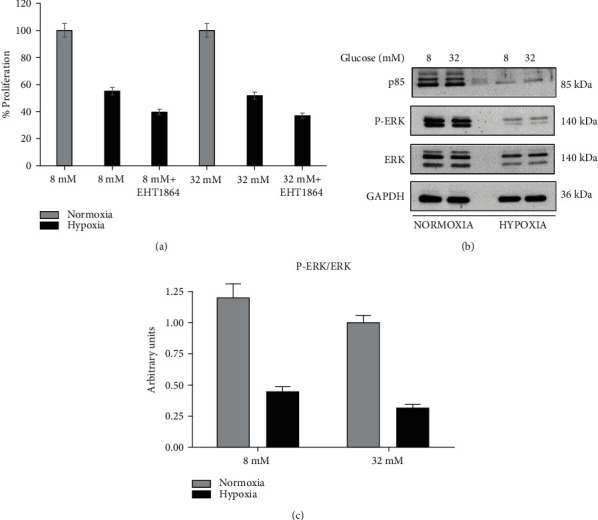
Cell viability assessed by MTT assay (a). The cells were pretreated with glucose (8 mM or 32 mM) for 15 min before undergoing a hypoxic insult for 24 h. For analysis of the effect of EHT 1864, cells were preincubated with EHT 1864 (20 *μ*M) for 30 min, and after, they were incubated in the presence of glucose (8 and 32 mM). The data are presented as the mean ± standard deviation of three independent experiments. (b) Lysate proteins (1 mg/ml) were resolved by electrophoresis and analyzed by Western blot, using the antibodies against the indicated proteins. The band intensity of p-ERK and ERK was determined by densitometric analysis using the image software (c).

Glucose metabolism plays an important role in cell survival. During ischemia, several changes are induced in cardiomyocyte metabolism, including a marked increase in glucose uptake and utilization. Here, we investigated whether acute glucose administration induces a cytotoxic effect in cardiomyocyte cell line H9c2, during hypoxic stress. We tested the effect of two glucose concentrations (8 mM and 32 mM) on the H9c2 viability by the MTT assay in normal and hypoxic conditions. The H9c2 cell exposure to hypoxia (24 hours) reduced cell viability to 50% compared to cells grown under standard conditions both at 8 and 32 mM glucose concentrations (Figure 1(a)). So, the acute glucose administration has not induced any effect. To evaluate whether EHT 1864, a Rac1 inhibitor, could affect cell viability, we pretreated H9c2 cells with EHT 1864 (20 *μ*M) for 30 min and then treated them with glucose (8 and 32 mM) under normal and hypoxic conditions. As shown in Figure 1(a), EHT 1864 significantly lowered cell viability by about 30%.

Since the PI3K/ERK pathway regulates multiple biological functions, including cell transformation, survival, differentiation, and apoptosis, and recently has emerged as a key player in cardiac physiology by improving contractility, we wondered if the glucose exposure and hypoxia could influence this signaling pathway in H9c2 cells. We observed PI3K regulatory subunit p85*α* expression levels decrease correlated to a lower ERK activation in H9c2 cells cultured under hypoxia and treated with 8 and 32 mM glucose compared to cells cultured under normoxia (Figures 1(b) and 1(c)) [48–50]. These results corroborate the hypothesis that hypoxia impacts cell viability.

**Figure 2:**
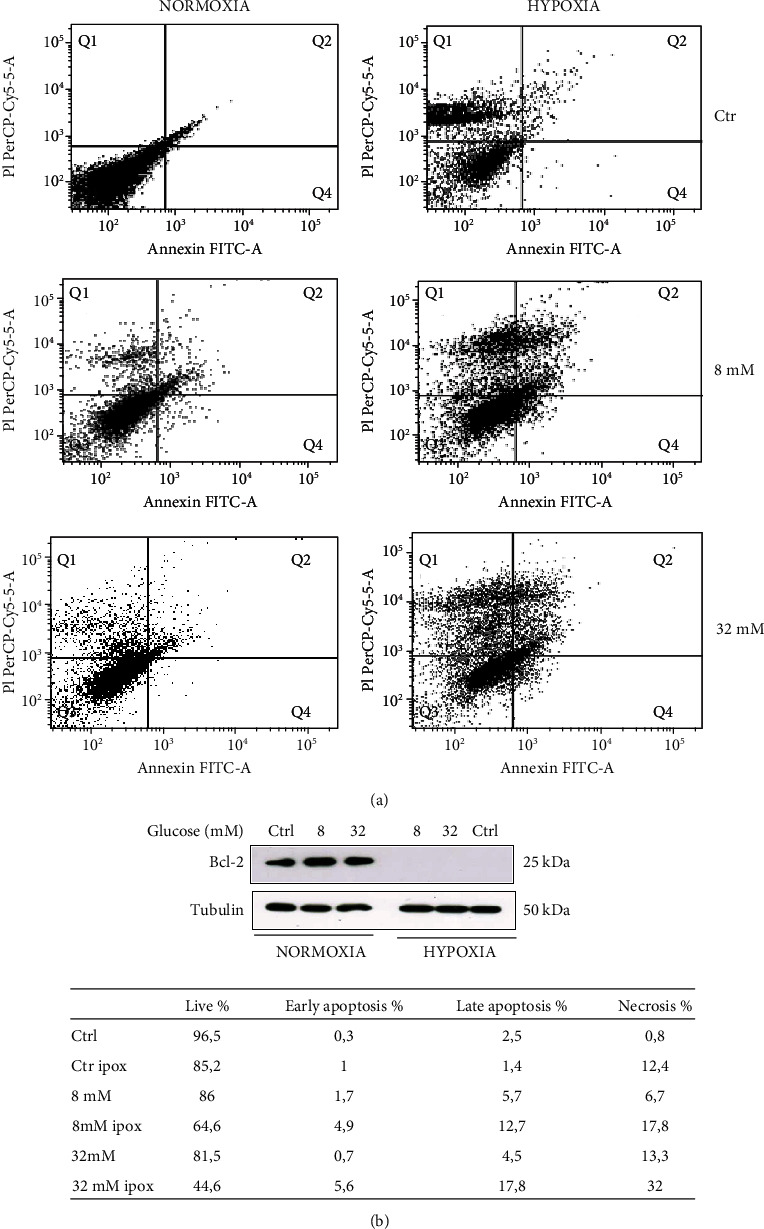
Apoptosis by annexin V-FITC/PI assay (b). Staining with annexin V or PI resulted in matching fractions of vital cells, early/late apoptosis, and necrotic cells. Cells in region Q3 represent living cells, cells in Q4 early apoptotic cells, cell in Q2 late apoptotic cells, and cells in Q1 those with a damaged membrane only. Each panel corresponds to a representative analysis of at least three separate experiments. (a) Western blotting analysis for Bcl-2 and tubulin in normalized lysates. Lysate proteins (1 mg/ml) were resolved by electrophoresis and analyzed by Western blot, using the antibodies against the indicated proteins. The table represents percentages indicated in the respective quadrants.

The annexin V-FITC/PI assay was employed to verify the hypoxia effect on apoptosis in H9c2 cells treated with both glucose concentrations. As shown in Figure 2(a), hypoxia reduced the cell viability of 8 and 32 mM glucose-treated H9c2 cells to 64.6 and 44.6%, respectively, compared with cells grown in normoxia. After hypoxic insult, the cells treated with 32 mM glucose showed a 2-fold significant increase of apoptosis compared to the cells treated with 8 mM glucose. In addition, Western blot analysis revealed that the Bcl-2 protein was undetectable after hypoxic insult (Figure 2(b)).

Collectively, these results revealed that hypoxia induced cell death and hyperglycemia worsened these effects (Figure 2(a)).

### 3.2. The Role of Rho/Rho-Kinase-Pathway in the H9c2 Cell Response to Hypoxia

**Figure 3:**
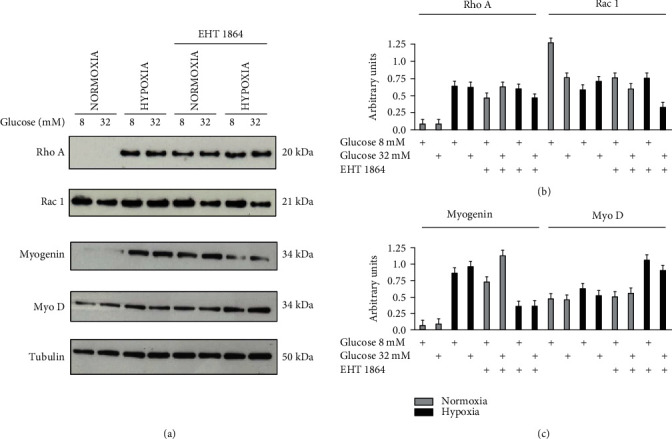
Western blotting analysis for RhoA, Rac1, myogenin, and MyoD in normalized lysates. Lysate proteins (1 mg/ml) were resolved by electrophoresis and analyzed by Western blot, using the antibodies against the indicated proteins (a). The band intensity was determined by densitometric analysis using the ImageJ software (b, c).

Since it has been reported that RhoA is activated in response to hypoxia in a variety of cell types including cancer cells [51], pulmonary artery smooth muscle cells [52], and ventricular myocytes [53], we aimed to evaluate the involvement of RhoA and Rac1 during hypoxic stress in cardiomyocytes (Figure 3). We observed that hypoxic insult induced RhoA expression level increase at both glucose concentrations (8 and 32 mM). When cells were treated with 8 mM glucose under hypoxia, we observed a lower expression of Rac1 compared to 32 mM glucose. The Rac1 expression increase correlates with the two stress conditions: hypoxia and high glucose, underlining that the glucose injury is mediated by Rac1. Moreover, during hypoxia, the glucose effect on Rac1 expression is more evident. So, we underline that the RhoA expression increase depends on hypoxia, while no differences are observed under the two glucose concentrations. We next assessed if inhibition of Rac1 by EHT 1864 affects other downstream signaling pathways responsible of the cytotoxic effect observed in H9c2. Data depicted in Figure 3 indicated a significant increase of the RhoA expression level under normoxia maybe to compensate for the Rac1 loss of function.

To determine whether hypoxia could influence the expression of myogenic regulatory factors critical for skeletal muscle development, such as MyoD and myogenin, we detected the expression of these proteins in cardiomyocytes by Western blot analysis. In both hypoxia and normoxia conditions, H9c2 cells did not show significant modification in the MyoD expression level. However, H9c2 cells cultured under hypoxia condition and treated with 32 mM glucose showed a significant reduction of myogenin expression compared to 8 mM glucose-treated H9c2 cells (Figure 3). MyoD improves myoblast formation and represents a factor of specification. Our results show that the MyoD level does not change under hypoxia in consideration that H9c2 cells are not able to differentiate. On the other hand, hypoxia induced myogenin expression, confirming, in agreement with other studies, its crucial role in cell regeneration [54, 55]. Furthermore, we examined the EHT 1864 ability to regulate the myogenic regulatory factor expression. We observed that myogenin expression decreases particularly in hypoxia with 8 and 32 mM glucose concentration. Taken together, these data suggest a specific role of hypoxia-induced Rac1 activation in mediating downstream signaling pathways.

### 3.3. Hypoxic Stress and NO Production in H9c2 Cells

**Figure 4:**
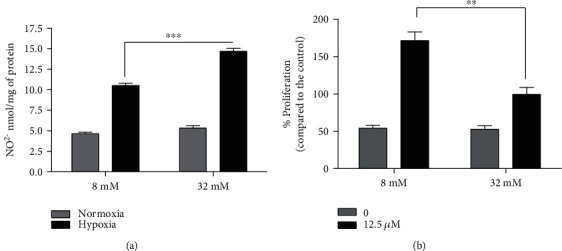
Nitrite ion levels by the Griess assay in the cell culture medium before and after hypoxia with 8 mM glucose or 32 mM glucose. In the bottom panel, H9c2 viability by MTT assay after 24 h pretreatment with 1400 W (12.5 *μ*M), iNOS inhibitor, and then stimulated with 8 mM or 32 mM glucose for 15 min before undergoing a hypoxic insult for 24 h.

ROS generation increases during ischemia-reperfusion, and it plays a pivotal role in the pathophysiology of intraoperative myocardial injury. NO is generated in cardiomyocytes by NOS, and it represents an endogenous regulator of myocardial function. Increased NO production, as a result of increased expression of iNOS, decreases myocardial function following myocardial infarction ischemia and dilated cardiomyopathy [56, 57]. Furthermore, iNOS expression was increased during sustained ischemia, and the concomitantly increased levels of nitrite/nitrous species are related to high iNOS activity and NO production [58]. NO is a relatively stable free radical that freely diffuses across cell membranes, reacting spontaneously with oxygen and water to form nitrite (NO_2_^−^). Therefore, we investigated if the pretreatment of H9c2 with different glucose concentrations modulates the hypoxia-induced NO_2_- production. In H9c2 cells cultured in hypoxia, we observed increased (about twofold) NO_2_- levels (Figure 4(a)) compared to the H9c2 cells cultured in normoxia. However, pretreatment with a high glucose concentration (32 mM) significantly increased the level of NO_2_^−^ during hypoxia compared to cells treated with 8 mM glucose. These results suggested that acute glucose exposure has a prooxidant effect in response to hypoxia in H9c2 cells. Based on these results, we investigated the role of iNOS by using 1400 W, a selective iNOS inhibitor in H9c2 cardiomyocyte proliferation. First, we performed a dose-response curve by administering the inhibitor at three different concentrations (12.5, 25, and 50 *μ*M) for 24 h (data not shown). As shown in Figure 4(b), the preventive administration of the 1400 W inhibitor, at the concentration of 12.5 *μ*M, increased significantly cell proliferation in hypoxia at both glucose concentrations. In addition, high glucose concentration (32 mM) induced a decrease in proliferation rate than low glucose treatment (8 mM). These results indicate that hypoxic stress causes cardiomyocyte damage through an increase of NO production by iNOS.

### 3.4. Cytoskeleton Morphology Changes and RhoA Expression under Glucose Treatment and Hypoxic Insult in H9c2 Cells

**Figure 5:**
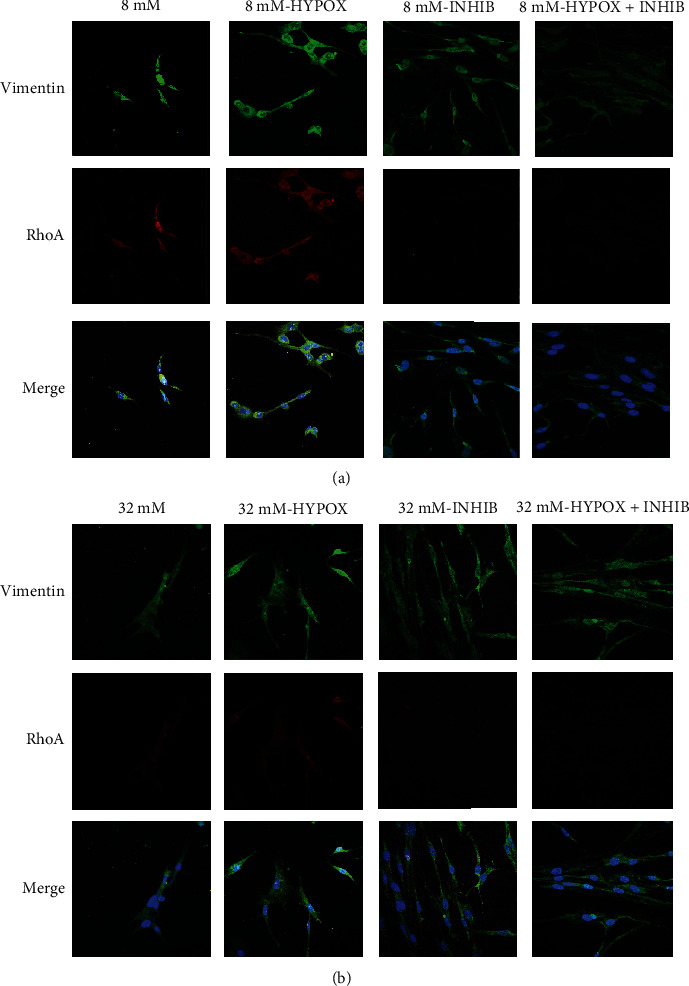
H9c2 cells were seeded on glass slides for 24 h. After the treatment with 8 mM (a) or 32 mM (b) glucose in both normoxia condition and hypoxia conditions, in the presence and absence of 1400 W (12.5 *μ*M), cells were stained as described in Materials and Methods. The analyses were performed with a Zeiss LSM 510 microscope equipped with a plan-apochromat objective X63 (NA 1.4) in oil immersion. 50 *μ*m scale bar is referred to all images shown.

In Figure 5, we reported the cytoskeleton morphology changes in H9c2 cells pretreated with 8 mM and 32 mM glucose in normoxia and hypoxia. The cells were fixed, stained with anti-vimentin antibody, and analyzed by confocal microscopy. The H9c2 cells after hypoxic insult acquired a rounded morphology shape; the H9c2 cells treated with 8 mM glucose appeared with a compact organization, while after the hypoxic insult, the cells assumed a plus disordered morphology. The H9c2 cell treatment with iNOS inhibitor induced morphology changes. Cells pretreated with both 8 and 32 mM glucose concentrations, before and after the hypoxic insult, showed an elongated shape with cell-cell contacts and a parallel organization.

In normoxia condition, under 8 mM glucose treatment, RhoA is absent according to physiological conditions, while under 32 mM glucose treatment, RhoA is localized both in the nucleus and in the cytoplasm (see Figure 3(a)). In the hypoxic condition, RhoA shows a low expression level under the 8 mM glucose treatment; the 32 mM glucose treatment induced an increase of RhoA expression that is localized in both the nucleus and the cytoplasm. In normoxia, for H9c2 cells treated with the iNOS inhibitor, RhoA is poorly expressed under the 8 mM glucose treatment and it is absent in the nucleus; under the 32 mM glucose, RhoA is localized in pseudopodia granulations. In hypoxia, under the 8 mM glucose treatment and in the presence of the iNOS inhibitor, RhoA is not expressed; while under the 32 mM glucose, RhoA has a low amount in pseudopodia granules and the rescue of the cell shape is evident (Figures 5(a) and 5(b)).

Subsequently, we investigated if EHT 1864, by inhibiting Rac1/ERK activation, elicits cell morphology changes. To address this point, H9c2 cells were pretreated with EHT 1864 and then incubated with glucose (8 and 32 mM) in normoxia and hypoxia, and finally, we examined alterations in cell morphology by confocal microscopy. Incubation of these cells under conditions of inhibition of cell signaling events did not lead to significant abnormalities in cell morphology (data not shown). These data indicate that EHT 1864 attenuates Rac1 activation without exerting significant effects on cellular events leading to abnormal cell morphology.

## 4. Conclusions

**Figure 6:**
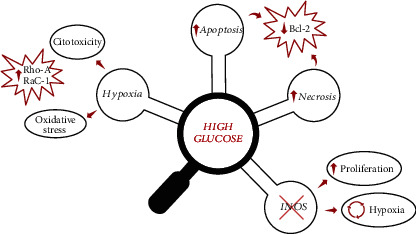
Biological effects of oxidative stress in high glucose-treated H9c2cells. The scheme shows an increase of cytotoxicity and oxidative stress and related effectors Rho-A/Rac1 and the downregulation of Bcl after exposure to high glucose. In addition, the preventive administration of the 1400 W iNOS inhibitor induces cell proliferation.

Focusing on the role of glucose pathways modulated by hypoxic stress, we report proliferation, apoptosis, oxidative stress, and cytotoxicity in cardiomyocytes, multiple biological effects regulated by class I phosphoinositide 3-kinases (PI3Ks), and its downstream target proteins. Here, investigations into the mechanism of oxidative stress correlate with an increase of expression of RhoA and Rac1 in the high glucose-treated H9c2 cells, confirming the crucial role of these proteins to drive regulated important biological processes (Figure 6). In this context, we evaluated the increase of the expression of myogenic regulatory factors such as MyoD and myogenin in hypoxia. Our evidences confirm previous studies focusing on the myogenin rescue after glucose treatment and hypoxic stress of H9c2 cells. Using confocal microscopy to control cytoskeletal dynamism, we have identified the cell shape mechanism mediated by RhoA activity specifically in H9c2 cells pretreated with two-glucose concentrations before a hypoxic insult. To eliminate ambiguities regarding the elongation shape and the parallel cell organization, we propose an interesting experimental approach to detect RhoA using the incubation of H9c2 cells with an iNOS inhibitor. In normoxia, RhoA is clearly present in pseudopodia granulation under glucose treatment, while RhoA is found under hypoxia and the iNOS inhibitor treatment induces the rescue of the cell shape. The use of a Rac1 inhibitor, EHT 1864, selectively inhibits Rac1 downstream signaling. In conclusion, the current study provides evidence linking the RhoA and Rac1 activation pathway to cardiac stress in the presence of glucose. The crucial role of these proteins in ischemia could represent novel sentinel targets useful for the clinical outcome monitoring. Further experiments performed on animal models are needed to evaluate the implication of these proteins in ischemic heart disease in the presence of glucose. Finally, the inhibition of the Rho/Rho-kinase-mediated signaling pathway could be a useful target therapy in patients at high risk of cardiovascular diseases such as diabetics. Therefore, these findings could represent a starting point to design new clinical trials for the treatment of ischemia.

## Data Availability

Answer: No. Comment: the data underlying the findings of our manuscript entitled “H9c2 Cardiomyocytes under Hypoxic Stress: Biological Effects Mediated by Sentinel Downstream Targets” are available from the corresponding author upon request (e-mail: antonia.feola@unina.it). Detailed information about results, material and methods to replicate the experiments, and conduct of secondary analyses is included within the manuscript.
